# Effect of temperature and humidity on insect DNA integrity evaluated by real-time PCR

**DOI:** 10.1093/jee/toae193

**Published:** 2024-08-30

**Authors:** Elizabeth V Fowler, Melissa L Starkie, Mark J Blacket, David G Mayer, Mark K Schutze

**Affiliations:** Department of Agriculture and Fisheries, Biosecurity Queensland, Brisbane, QLD, Australia; Department of Agriculture and Fisheries, Agri-Science Queensland, Brisbane, QLD, Australia; Department of Agriculture and Fisheries, Biosecurity Queensland, Brisbane, QLD, Australia; Agriculture Victoria Research, Invertebrate & Weed Sciences, AgriBio Centre, Bundoora, VIC, Australia; Department of Agriculture and Fisheries, Agri-Science Queensland, Brisbane, QLD, Australia; Department of Agriculture and Fisheries, Biosecurity Queensland, Brisbane, QLD, Australia

**Keywords:** biosecurity, DNA preservation, gBlock, Tephritidae

## Abstract

Insects collected in dry traps can degrade rapidly, especially in warm, humid environments where many biodiversity and biosecurity surveillance activities are undertaken. Degradation can severely impact diagnostics, as trap catches can become difficult to identify to species level using morphological characters or, of increasing importance, molecular approaches. This is especially problematic for biosecurity surveillance of exotic tephritid fruit flies, where diagnostics are heavily reliant on morphological characters. We tested the effects of differing temperature and humidity conditions on mock samples of tephritid fruit flies in a controlled environment and compared our results to field trap catches. DNA degradation was quantified using real-time PCR assays, including one assay newly developed and tested here. We observed a correlation between increasing DNA degradation and increasing temperature and humidity. The greatest DNA degradation occurred under combined high humidity (90% relative humidity) and constant high temperature (35 °C). Unexpectedly, fluctuating temperature did not have a significant impact on DNA. Other factors, such as trap design, time in the field, and rainfall, did not significantly correlate with DNA quality across the field samples tested. When plotted against mock samples, field samples clustered together, with no clear pattern or predictability regarding the quantity of DNA preserved, indicating other untested environmental variables may be at play. Predictably, increased exposure time was found to have a detrimental effect on DNA quality for all treatments. These findings will improve the delivery of surveillance activities through the implementation of shorter trap clearance timeframes and improved trap designs and procedures.

## Introduction

Insect surveillance programmes can serve many functions (i.e., biodiversity and biosecurity), but their primary output is the presence and/or absence of data for taxa in environments of interest. Biosecurity agencies generate absence data for exotic insect pests largely through the deployment of trapping grids (Department of Agriculture, Fisheries and Forestry 2023). The success of exotic insect surveillance programmes is largely dependent on the condition of field-trapped samples received by the diagnostic laboratory. Samples are often obtained through passive trapping systems that are monitored at frequent intervals (Bashford 2008), and it is the period between collecting intervals that samples are susceptible to degradation and damage from the elements (Lindahl 1993, Zimmermann et al. 2008). Exposure to environmental variables, such as water, UV radiation, pH, and salts, can impact diagnostic protocols and their success (Seutin et al. 1991, Lindahl 1993, Walker and Sikorska 1994, Dessauer et al. 1996, Dean and Ballard 2001, Mandrioli et al. 2006, Ballare et al. 2019). For morphological methods to succeed, samples with minimal damage or discoloration to their diagnostic characters are preferred, while molecular diagnostics requires samples with minimal exposure to mold and decay, which may cause DNA degradation (Martoni et al. 2021).

To mitigate specimen degradation, surveillance programmes may use “wet traps” that include a preservative; however, wet traps come with their own challenges (Boykin et al. 2014, Martoni et al. 2021, 2023). For example, propylene glycol is a commonly used preservative, but its use may require additional treatment of samples (e.g., washing and sample filtration) prior to morphological examination or molecular diagnostics (Martoni et al. 2023). There are also some documented instances of propylene glycol negatively impacting morphological diagnostic characters (Plant Health Australia 2018) and molecular-based diagnostic methods (Ballare et al. 2019). For large surveillance activities, liquid preservation methods introduce additional challenges, such as increased time to clear and recharge traps and discoloration of morphological characters (Clarke 2019). The alternative, “dry traps” without preservatives, are widely used in many surveillance programmes, especially where they must conform to stringent regulatory guidelines for pest species such as dacine tephritids, e.g., International Standards for Phytosanitary Measures 26: Establishment of pest-free areas for fruit flies (FAO/IPPC 2006). The question remains, however: if dry traps must be used, how long can samples remain in the field before they are unsuitable for molecular diagnostics?

Dacine fruit flies (Tephritidae: Dacinae) are a diverse group of insects principally distributed across tropical and subtropical Asia, Australia, and the Pacific. Representing over 900 species (Doorenweerd et al. 2018), dacines comprise mostly nonpest species, but some are global horticultural pests, such as the Oriental fruit fly, *Bactrocera dorsalis* (Hendel) and melon fly, *Zeugodacus cucurbitae* (Coquillett) (Clarke et al. 2005, De Meyer et al. 2015, Schutze et al. 2015). Crucially, these pests have different yet overlapping geographic distributions and, as such, do not all occur in the same countries or regions (Plant Health Australia 2018). Consequently, they are the targets of intensive regional biosecurity surveillance programmes designed to detect early incursions to improve prospects for a successful eradication response.

Biosecurity surveillance programmes are reliant not only on morphological identification but also on genetic approaches for diagnostics (Piper et al. 2019). Due to the high volume of samples trapped during each clearance period (which can number in the thousands), improving turnaround times with complementary molecular approaches are favorable. Given that these approaches require samples in reasonably good condition, an understanding of how temperature and humidity can affect tephritid fly quality is critical.

The aims of this study were to: (i) expose colony-reared tephritid flies to various temperature and humidity combinations to identify the factors that most affect DNA quality; and (ii) compare our findings to wild dacine flies collected from a range of environments representing variability in temperature and humidity. Our overarching goal for this research is to inform trap deployment and clearance timeframes to improve sample quality for both morphological identification and for high throughput molecular diagnostic approaches. The ongoing improvement and evolution of wide-scale surveillance of dacine tephritids in Australia and other regions will be supported by this work.

## Materials and Methods

### Samples

Colony tephritid flies were sourced from *Bactrocera tryoni* (Froggatt), *Bactrocera neohumeralis* (Hardy), *Bactrocera jarvisi* (Tryon) and *Zeugodacus cucumis* (French) cultures maintained by the Queensland Department of Agriculture and Fisheries (QDAF) facilities located at Brisbane and Cairns, Queensland (Australia). Flies were killed by freezing at −20 °C. We compiled mock tephritid fly samples for testing, consisting of ~500 *B. tryoni* (by weight ~3.5 g) spiked with either 5 *Z. cucumis* (treatments 1 and 2) or 1 *Z. cucumis* and 1 *B. jarvisi* (treatments 3–7) (more detail below) as proxies for an “exotic detection” to test the limit of detection of the real-time PCRs and simulate detection of low numbers within a larger sample.

Wild-caught dacine species flies were obtained from 30 field traps cleared between October 2020 and November 2021 (Supplementary Table 1). Traps were maintained by Biosecurity Queensland, QDAF, as part of ongoing Biosecurity Queensland and “National Plant Health Surveillance Program” surveillance in line with ISPM 26 (FAO/IPPC 2006). Three dry trap designs are commonly used by the program depending on the trapping location Lynfield (Cowley et al. 1990), Paton (Huxham 2002), and Modified Steiner (Hooper and Drew 1978) (Supplementary Table 1), which are baited with Dacine-specific lures (Clarke 2019). A subset of ~500 mixed dacine flies (by weight ~3.5 g) were sampled from each trap and stored at −20 °C for real-time PCR analysis.

### Mock Samples Subjected to Controlled Temperature and Humidity

Mock tephritid fly samples were evenly spread in a lidless plastic container (12 cm wide × 5.5 cm high × 17 cm deep), which was placed in a controlled temperature and humidity cabinet (Humiditherm TRH-460; Thermoline Scientific, Sydney, Australia). We undertook 7 treatments using variables that represented a variety of subtropical and tropical conditions based on averaged Australian Bureau of Meteorology temperature and humidity data for southeast and northern Queensland (Supplementary Table 1) (Australian Bureau of Meteorology 2022). Treatments 1–5 subjected flies to constant temperature and humidity conditions for the duration of the trial, while treatments 6 and 7 maintained constant humidity but experienced fluctuating temperatures over the same period (see Lee et al. (2019)) (Table 1). Temperature and humidity were monitored during experiments using a HOBO U23-001 Pro v2 Temp/RH Data Logger (OneTemp, Australia) to ensure consistency between the cabinets and experiments and to confirm that the cabinet settings generated the required conditions. Cabinet temperature and humidity data recorded during experiments demonstrated little deviation from programmed temperature parameters (see Supplementary Table 2). For each treatment, 5 containers were removed from the cabinet after 2 wk (A), while 5 remained for the full 4 wk (B). One variation to this schedule occurred in treatment group 3 due to extensive mold contamination in the cabinet chamber, for which the trial was halted at 2-wk (3A) and the cabinet was decontaminated. Insufficient flies were available at the time to repeat both time points, so only the longer duration was repeated; however, this was terminated at 3-wk (3B) due to a cabinet failure. Following the treatments, samples were stored at −20 °C before DNA isolation and real-time PCR analysis. Three additional samples (also consisting of ~500 *B. tryoni*, 5 *Z. cucumis*, and 1 *B. jarvisi*) that did not undergo any treatment were stored at −20 °C before DNA isolation and then processed as untreated controls.

**Table 1.** Treatment variables used in the mock sample are constant temperature and humidity trials (1–5) and fluctuating temperature, stable humidity cabinet trials (6 and 7). Ten replicate samples/treatment; subsample A = 2-wk trial (5 replicates removed after 2 wk); subsample B = 4-wk trial (5 replicates remained for the full 4 wk). Note: Extensive mold growth was observed in the cabinet chamber during treatment 3A. Cabinet failed at 3-wk during treatment 3B

Treatment 1	Treatment 2	Treatment 3	Treatment 4	Treatment 5	Treatment 6	Treatment 7
50% Humidity	70% Humidity	90% Humidity	50% Humidity	90% Humidity	70% Humidity	70% Humidity
20 °C constant	27.5 °C constant	35 °C constant	35 °C constant	20 °C constant	25–30 °C cycling: 3 h @ 26.5 °C; 3 h @ 28.5 °C; 6 h @ 30 °C; 3 h @ 28.5 °C; 3 h @ 26.5 °C; and 6 h @ 25 °C	22.5–32.5 °C cycling: 3 h @ 25.82 °C; 3 h @ 29.14 °C; 6 h @ 32.5 °C; 3 h @ 29.14 °C; 3 h @ 25.82 °C; and 6 h @ 22.5 °C

500 flies, 10 replicates for each treatment, subsampled at 2 and 4 wk, with lights off.

### DNA Isolation and Evaluation by Real-Time PCR Assays

Crude DNA was isolated from the mock samples and field samples using a nondestructive method as described in Fowler et al. (2023). Briefly, flies were gently submerged in 10 ml of HotSOAK buffer (12.5 mM NaOH; 5 mM Tris-HCl; 0.5 mM EDTA, pH 8.0) and incubated at 75 °C for 10 min. An aliquot of lysate (~1 ml) was stored at −20 °C for further analysis. Equipment was cleaned between each treatment group to avoid any potential cross-contamination.

DNA quality was evaluated by 4 real-time PCR assays: 3 species-specific assays targeting *Z. cucumis* (Li et al. 2019), *B. jarvisi* (Li et al. 2019), and *B. tryoni* (assay as per Dhami et al. (2016), cycling conditions as per (Blacket et al. 2020)) targeted the mock samples, while a Dacine-COI PCR (Krosch et al. 2020) was modified here to: (i) quantify total DNA from the mixed field samples; and (ii) avoid amplification of mold and other contaminants. Modifications to the species-specific assays and reaction conditions are detailed in Fowler et al. (2023). The Dacine-COI real-time assay used PCR primers designed for dacine tephritids (Krosch et al. 2020) (for reaction conditions, efficiency, and limit of detection, see Supplementary Information). Reactions containing no template were included to control for potential PCR assay contamination.

Four custom-made gene fragments synthesized as double-stranded (ds) DNA gene Blocks (gBlocks; Integrated DNA Technologies, Iowa, USA) were designed as part of this study (Supplementary Table 3) and used as positive controls in each real-time assay. The gBlocks were designed corresponding to the 5ʹ end of the cytochrome c oxidase subunit 1 (COI) gene, with additional strings of -ggg- or -ccc- sequences inserted to increase melting temperature (Tm) so that it could be distinguished from true PCR product as determined by in silico PCR using uMelt Quartz Melting Curve Predictions Software, Build 3.6.2; https://dna-utah.org/umelt/quartz/ (Dwight et al. 2011). Copy number of all gBlocks was determined using an online calculator (SciencePrimer, http://sciprim.com/html/copyNumb.v2.0.html), and gBlocks were resuspended in nuclease-free water at 10^10^ copies/μl as per manufacturer’s instructions. Serial 10-fold dilutions of gBlocks were prepared down to 10 copies/μl in nuclease-free water for evaluation of assay performance, quality control between runs, and to prepare an external calibration curve.

All real-time PCR assays were run on a Rotor-Gene RGQ Real-time PCR cycler (Qiagen, Australia). DNA from the untreated control samples were evaluated in all 4 real-time PCR assays. Copy number was determined by the Rotor-Gene Q software (version 2.3.5) with reference to the external calibration curve. For comparison purposes, Dacine-COI assay data were adjusted for 10^3^-fold decreased assay sensitivity relative to the *B. tryoni* probe-based assay.

### Data and Statistical Analyses

Data values were highly skewed with heterogeneous variances, so were analyzed using generalized linear models (McCullagh and Nelder 1989) in Genstat v21.1 (2021). The Gamma distribution with the log link function was the most appropriate for these data and was adopted. The Ct values from the species-specific real-time PCR assays (*B. tryoni*, *Z. cucumis*, and *B. jarvisi*) for all 7 treatments were analyzed as 7 discrete levels using a regression analysis and Fisher’s protected LSD-testing at the *P* < 0.05 level. For all 3 species-specific datasets, we tested the fitted factors: treatment, weeks (subjected to treatment), treatment~weeks. The Dacine-COI real-time PCR data from the field-caught flies were tested with the following fitted factors: collection locality, weeks (in the field), temperature, humidity, rainfall, and trap design. Data on temperature, humidity, and rainfall for the field trap catches were averaged and taken from Australian Bureau of Meteorology (2022) (see Supplementary Table 4). In order to compare mock and field trap catch data across the *B. tryoni* real-time PCR assay and the Dacine-COI real-time assay, we calculated DNA copy number using an online calculator (SciencePrimer, http://sciprim.com/html/copyNumb.v2.0.html).

## Results

### Factors Affecting DNA Integrity of the Mock Samples

**Fig. 1.**
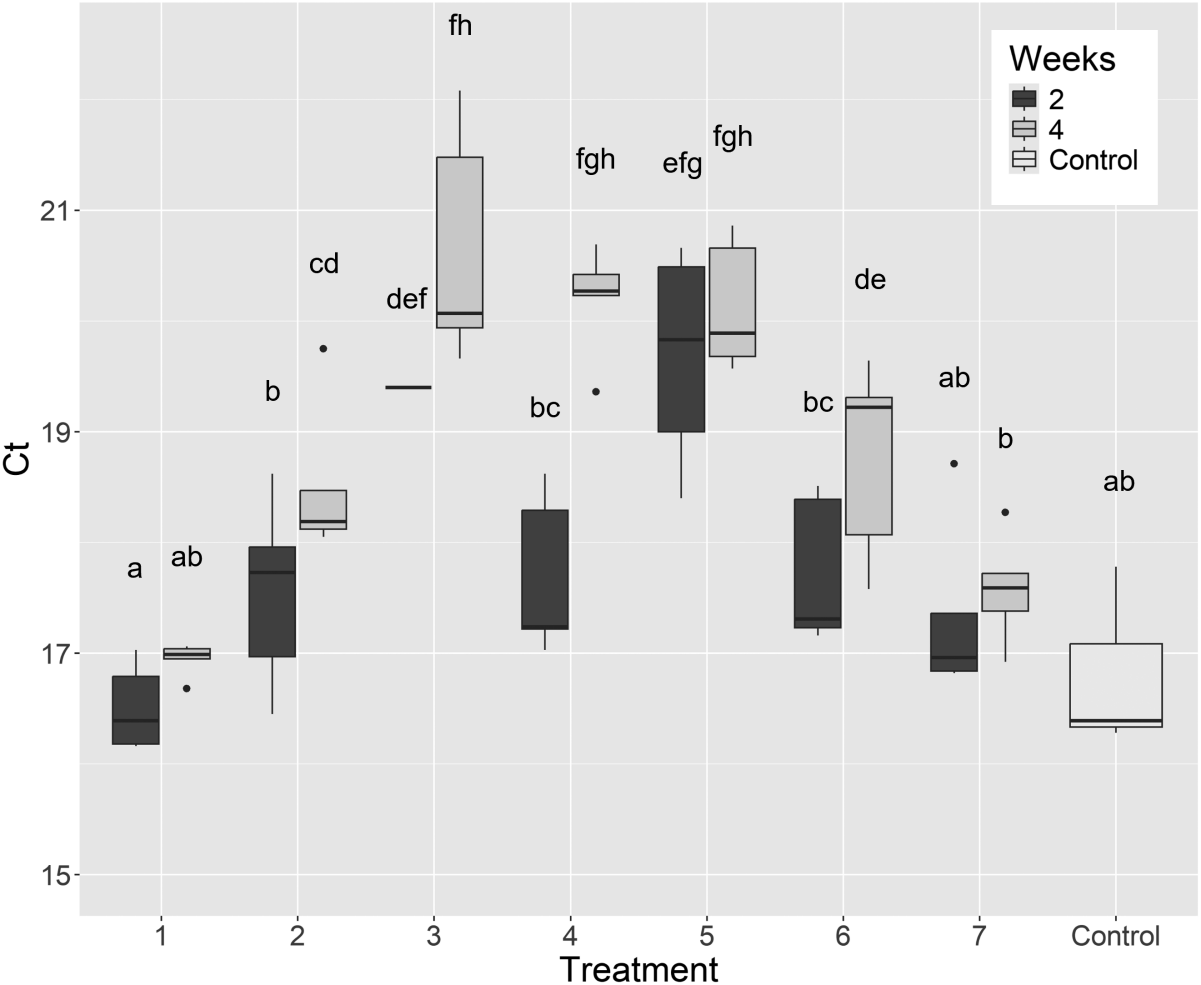
Comparison of *Bactrocera tryoni* real-time PCR results (adjusted mean Ct values) from 7 colony-fly treatments at two-time points and an untreated control. There was a significant difference between treatment groups where the letters above are different (*F* = 16.33; *df* = 14, 54; *P* < 0.001) and means sharing a common letter are not significantly different. Five constant temperature and humidity treatments were (i): 20 °C/50% RH; (ii): 27.5 °C/70% RH; (iii): 35 °C/90% RH*; (iv): 35 °C/50% RH; (v): 20 °C/90% RH. Two fluctuating temperature treatments were (vi): 25–30 °C/70% RH; (vii): 22.5–32.5 °C/70% RH. Two-time points at A: 2 wk; B: 4 wk. See Table 1 for precise fluctuating temperature regimens (*one data point only for treatment 3A as samples failed to amplify; cabinet failed at 3 wk for treatment 3B).

While DNA was amplified in the *B. tryoni* real-time PCR for all treatments, the accumulated analysis of variance revealed there was a significant difference in Ct values for the *B. tryoni* assay among the 7 treatments (*F* = 16.33; *df* = 14, 54; *P* < 0.001). There was a general trend of increasing Ct value (i.e., decreasing DNA) as the temperature and humidity increased (Fig. 1). The lowest mean Ct value (highest DNA) was for the 20 °C/50% RH (1A) treatment at 2 wk, while the highest mean Ct value (lowest DNA) resulted from the 35 °C/90% RH (3B) treatment at 3 wk (Supplementary Table 5). The highest Ct values resulted from the 3 treatments that were subjected to either high temperature (4), high humidity (5) or both (3) in the *B. tryoni* data set. The accumulated analysis of variance revealed the factors treatment (*F* = 28.69; *df* = 6, 52; *P* < 0.001) and weeks (*F* = 28.15; *df* = 1, 52; *P* < 0.001) significantly impacted Ct values. When those factors were fitted together (treatment~weeks), they were also significant (*F* = 2.82; *df* = 6, 52; *P* < 0.05), indicating an interaction between time and humidity and temperature exposure.

Species-specific real-time PCR assays amplified DNA for *Z. cucumis* and *B. jarvisi* for all treatments except 35 °C/90% RH at 2 wk (3A) (Table 2). This sample had a high level of mold contamination on the flies when the treatment was terminated. The longer 35 °C/90% RH treatment (3B) amplified *B. jarvisi* DNA, but not *Z. cucumis*. The *Z. cucumis* and *B. jarvisi* mean Ct values were several cycles higher than any of the *B. tryoni* Ct values (Table 2). The accumulated analysis of variance found that across all treatments, there was no significant difference (*P* > 0.05) among *B. jarvisi* real-time PCR Ct values across all treatments. Conversely, treatment was found to have a significant effect on Ct values when analyzed against the *Z. cucumis* real-time PCR data (*F* = 38.96; *df* = 12, 30; *P* < 0.001). The factors weeks (*P* > 0.05) and treatment~weeks (*P* > 0.05) did not have a significant effect on *Z. cucumis* real-time PCR Ct values (Table 3).

**Table 2.** Adjusted average *Zeugodacus cucumis* and *Bactrocera jarvisi* species-specific COI real-time PCR data for 7 and 5 treatment groups, respectively, and collected at two-time points. *Z. cucumis* means with a common letter are not significantly different at *P* = 0.05; The *B. jarvisi* means were not significantly different at *P* = 0.05. N/A denotes no *B. jarvisi* in these mock sample treatments; *No data analyzed for group 3 when Ct > 40 **one data point only for treatment 3 at 4 wk (3B) as samples failed to amplify (Ct > 40)

Treatment	Temperature (°C)	RH (%)	Mean Ct values			
			Zeugodacus cucumis		Bactrocera jarvisi	
			2 wk	4 wk	2 wk	4 wk
1	20	50	23.87^a^	22.55^a^	NA	NA
2	27.5	70	23.96^a^	24.11^a^	NA	NA
3*	35	90	>40	>40	>40	37.21**
4	35	50	25.84^a^	24.63^a^	32.83	35.87
5	20	90	32.77^b^	31.05^b^	31.59	34.00
6	25.0–30.0	70	25.87^a^	26.21^a^	31.59	31.50
7	22.5–32.5	70	23.83^a^	26.11^a^	30.55	32.44
Untreated	N/A	N/A	23.34^a^	–	34.15	–
Pooled Standard Error			1.5	–	2.13	–

**Table 3.** Adjusted *B. tryoni* and *Z. cucumis* mean Ct values (species-specific COI real-time PCR) for 7 different treatment groups with temperature, humidity, and time as cofactors. Means with a common letter are not significantly different at *P* = 0.05; *No data analyzed for group 3 as Ct > 40

Treatment group	Temperature (°C)	RH (%)	Bactrocera tryoni	Zeugodacus cucumis
			Adjusted Ct value	Adjusted Ct value
1	20.0	50	16.74^a^	23.21^a^
2	27.5	70	18.06^bc^	24.03^a^
3	35.0	90	20.06^e^	*
4	35.0	50	19.01^d^	25.23^a^
5	20.0	90	19.92^e^	31.91^b^
6	25.0–30.0	70	18.27^c^	26.04^a^
7	22.5–32.5	70	17.46^b^	24.97^a^
Pooled Standard Error			0.25	1.03

### Real-Time PCR Analysis of Field Trap Catch Flies

The Dacine-COI real-time PCR results from field trap catch samples were variable (Supplementary Table 4). The regression analysis applied to the Ct values found no significant difference among any of the field trap catch samples (Table 4). The factors collection locality (*P* > 0.05), weeks (*P* > 0.05), temperature (*P* > 0.05), humidity (*P* > 0.05), and trap design (*P* > 0.05) did not significantly affect Ct values of the Dacine-COI real-time PCR. This is likely due to the little variation in average humidity and temperature at each field site. Average rainfall data was quite variable, and this was reflected in the regression analysis, where rainfall was found to be near-significant (*P* = 0.053).

**Table 4.** Environmental data (temperature, humidity, and rainfall) and mean Dacini COI real-time PCR data (Ct values) for all trap catches grouped according to region, time, and trap type. Untreated group (mock trial): mean Ct value = 25.58 ± 4.56. *Lynfield traps were mainly deployed (7/8) in the south and mostly collected at 2 wk (7/8), while Steiner and Paton traps were exclusively deployed in the north. All of the Steiner traps were collected at 2 wk, while the Paton traps were more frequently deployed for 4 wk (11/17)

Group*	No. traps	Temperature (°C)	Humidity (%)	Total rainfall (mm)	Ct values
All traps	30	30.77 ± 1.57	61.48 ± 6.65	87.92 ± 89.93	25.32 ± 4.81
North QLD	23	31.31 ± 1.25	63.25 ± 6.36	102.21 ± 95.55	24.99 ± 4.20
South QLD	7	28.99 ± 1.17	55.66 ± 3.67	40.97 ± 47.53	26.27 ± 6.57
Two weeks	18	30.77 ± 1.66	60.07 ± 6.75	65.26 ± 80.70	26.17 ± 5.55
Four weeks	12	30.77 ± 1.50	63.59 ± 6.16	121.92 ± 95.68	23.88 ± 2.92
Lynfield	8	29.62 ± 2.10	55.95 ± 3.50	37.43 ± 45.14	25.62 ± 6.35
Paton	17	31.44 ± 1.05	63.04 ± 6.56	101.75 ± 92.55	25.63 ± 4.59
Steiner	5	30.32 ± 1.00	65.00 ± 6.30	121.68 ± 116.06	23.55 ± 1.90

### Comparison Between Mock Samples and Field Catches

**Fig. 2.**
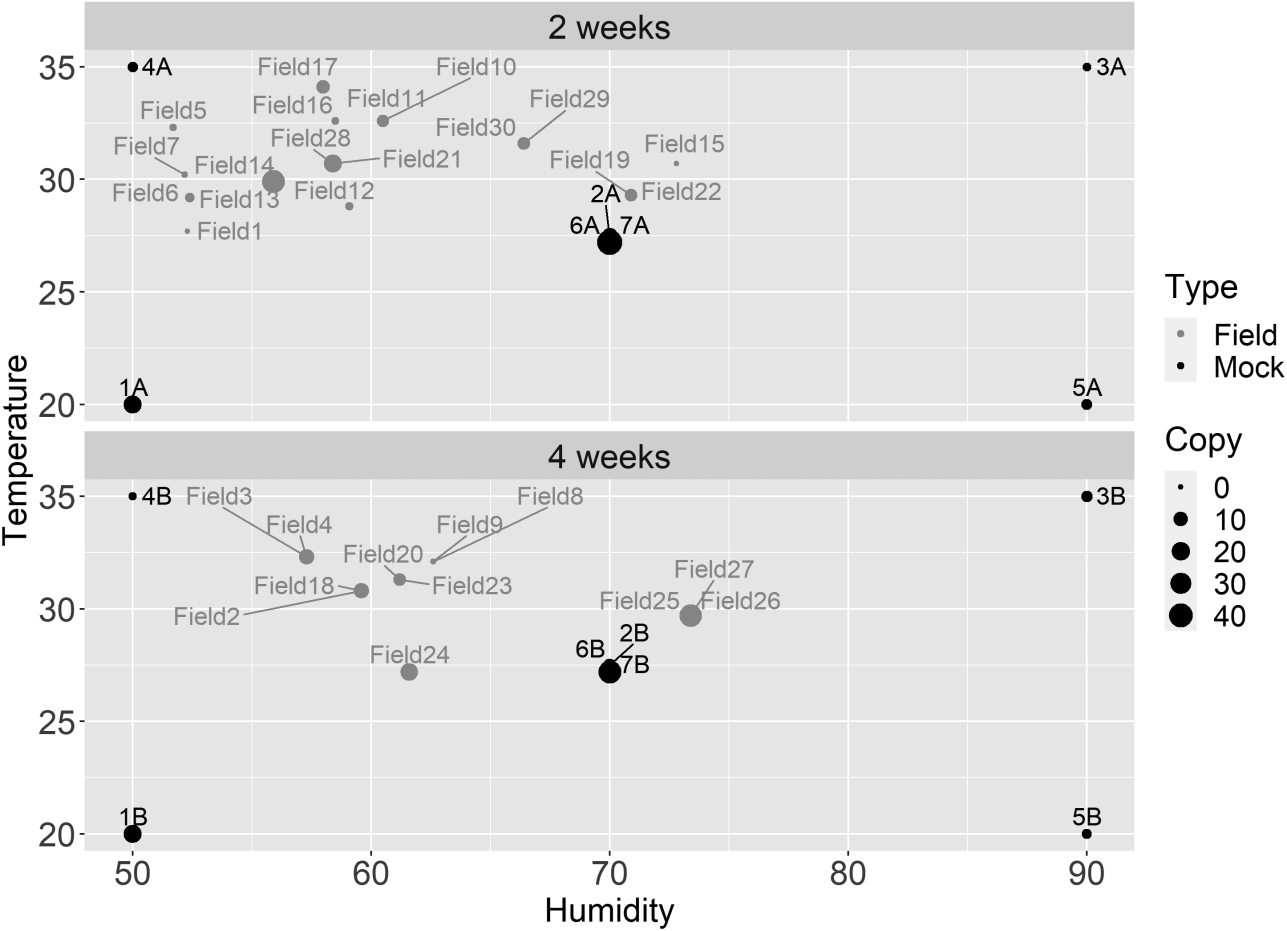
Comparison of average copy number data from mock samples determined by *B. tryoni* real-time PCR and copy number of field samples determined by Dacine-COI real-time PCR. Copy number data points increase with size; size guide provided as example size measures in legend. Note 1: data points Field1, Field2, Field9, Field15, and Field19 Ct > 39, copy number ≅ 0. Note 2: Field25, Field26, Field27 represent samples in the field for 3 wk.

The field catch real-time PCR results showed only 4 field samples with high-quality DNA (i.e., Dacine COI copy number > 7,000/ reaction), which were Field14 (2 wk, 30 °C, 56% RH); Field26 (4 wk, 30 °C, 73% RH); Field24 (4 wk, 27 °C, 62% RH) and Field28 (2 wk, 30 °C, 58% RH) (Supplementary Table 4). When adjusted for the difference in assay sensitivity, these results were comparable to the *B. tryoni* copy number results for the mock sample groups 1: 20 °C/50% RH at 2- and 4-wk; 2A: 27.5 °C/70% RH at 2 wk; and 7: 22.5–32.5 °C/70% RH at 2- and 4-wk. In contrast, there were 14 field samples (Field1, 2, 5-9, 11, 12, 15, 16, 19, 20, and 25) with low-quality DNA (i.e., Dacine COI copy number < 1,000/ reaction) collected at 28–33 °C, 52%–73% RH, 2 or 4 wk (Supplementary Table 4). These results were comparable to mock treatment groups 4B: 35 °C/50% RH at 4-wk and 3A: 35 °C/90% RH at 2 wk. The remaining field samples had moderately good DNA quality and were collected at 29–34 °C, 56%–66% RH, 2 or 4 wk (Supplementary Table 4). However, when DNA copy number results were compared, the adjusted field catches clustered with their similar average temperature and humidity ranges (Fig. 2).

## Discussion

### High Temp and Humidity Had a Negative Impact on DNA Quality

The DNA quality of tephritid flies from the controlled laboratory trials was negatively affected by higher temperature and humidity conditions, as well as longer exposure time. This is consistent with previous studies that demonstrated environmental factors, including high temperature and high humidity, are more likely to degrade DNA from insect specimens (Mandrioli 2008, Zimmermann et al. 2008), particularly if they are not collected directly into preservatives (Ballare et al. 2019). Others have shown that amplifiable DNA can persist in the environment for long periods on a variety of surface types, with DNA degradation or persistence critically dependent on high relative humidity and abundant rainfall (Lee et al. 2019). The detrimental effects of high temperature and/or high humidity on DNA quality were clearly apparent when sample material was limited. We also found that longer exposure times resulted in lower-quality DNA, but not across all treatments. These results are consistent with previous studies that found DNA quality is affected by storage temperature, relative humidity, and exposure time (Lund and Dissing 2004, Stevens et al. 2011, Martoni et al. 2021, Butterwort et al. 2022).

### Effects of Fluctuating Temperatures

Results for both fluctuating temperature treatments (6 and 7) were not statistically different from the control group or other low temperature and humidity treatments (e.g., 20 °C/50% RH treatment 1); with the exception of treatment 6B (25–30 °C; 70% RH; 4 wk). In addition, despite treatment 6B generating a higher average Ct, it was still relatively low (Ct = 18.76), thus demonstrating DNA of sufficiently high quality for amplification. When comparing treatments 6 (25–30 °C; 70% RH) and 7 (22.5–32.5 °C; 70% RH), there was a significant difference in Ct values of treatment 6 over 4 wk performed significantly worse than treatment 6 at 2 wk and treatment 7 at 2- and 4-wk. This could be due to the longer exposure time and the increased time spent at a higher temperature. Several studies have identified relative humidity as a critical factor in DNA preservation, where the lower the humidity, the better quality DNA can be recovered from a sample (Stevens et al. 2011, Lee et al. 2019). Consistent with our findings, Lee et al. (2019) found that even when the temperature varied in controlled environments (i.e., indoors), high-quality DNA preservation was possible, particularly when relative humidity was well-controlled. Therefore, while we found that temperature and humidity were co-factors that contribute to poor-quality DNA, it is likely that insect DNA is more sensitive to high humidity exposure.

### DNA Quality From Field Trap-Catch Flies Was Highly Variable

Dacine flies collected from the field demonstrated greater variability in DNA quality as compared to those from the controlled laboratory treatments. While wild-caught flies experienced variable lengths of time between capture and DNA extraction, we believe exposure to additional environmental conditions affected DNA quality. Similar to our study, Lee et al. (2019) produced variable field results but stable laboratory results when investigating degradation of DNA from various source materials exposed to tropical conditions. Lee et al. (2019) also controlled for temperature and humidity in the laboratory, suggesting other variables impacted the field results. UVB (Li et al. 2002) and UVA radiation can degrade DNA (Ravanat et al. 2001), and alongside humidity and temperature, sun exposure was found to have the highest impact on DNA quality of bird feathers (Vili et al. 2013), which corroborates our findings, which suggests sun exposure could have influenced our field results. Further work in this area may look to test the effects of UV rays and how they permeate the clear plastic used to construct fruit fly traps.

We observed a trend of lower DNA quality in samples caught in Paton and Lynfield trap designs. While this result was not significant or a factor we had initially set out to test, with greater sampling (our sample size *n* = 30), a stronger correlation might become evident. (Brown et al. submitted) found an effect of fruit fly trap design on water entry into traps. Observations of water entry into traps were also linked to degraded DNA and were evident in the Lynfield traps (Brown et al. submitted). It is possible that our lower-quality DNA is a result of direct water exposure in field traps, given the known impacts of water on DNA quality (Vili et al. 2013, Nakahama et al. 2019, Martoni et al. 2021).

### Detection of Low Abundant Species

The real-time PCR results demonstrated the capacity of the designed assays to detect species present in low abundance; however, some field catches failed to amplify DNA within the cycle threshold. Here, we reported average results of field temperature and humidity conditions for the period that flies were in the field, but this does not capture whether temperatures remained steady around the average or whether samples were exposed to high spikes in temperature or humidity during this period. All 5 samples that performed poorly experienced high average temperature, high relative humidity, and rainfall, while 2 of these field samples were exposed to these conditions for 4 wk. It is possible that exposure time and exposure to moisture contributed to poor-quality DNA. Further testing would be required to isolate the exact cause of DNA decay in the field. If the diagnosis of insects from these traps were to transition to molecular methods, operational adjustments might be required; for example, more frequent clearance schedules and improved trap designs to better protect samples from exposure to environmental variables.

### Impact and Future Directions

Our study represents the first thorough evaluation of the impact of 3 key abiotic environmental variables (temperature, humidity, and time) on the preservation of tephritid fruit flies in the Australian surveillance system. A better understanding of the impact of time on the degradation of samples under high temperature and humidity conditions will directly inform the design of surveillance programs and clearance schedules in tropical locations. In turn, this will mitigate the effects of temperature and humidity we identified and allow for seamless adoption of high throughput molecular approaches. Our findings could be extrapolated to inform trap surveillance timeframes for other insect groups (in dry traps), but further research may be required to determine optimal conditions for trapping and downstream molecular processing of sclerotized insects.

## Supplementary data

Supplementary data are available at *Journal of Economic Entomology* online.

toae193_suppl_Supplementary_Tables_S1-S5
