# Supplementary material for: Effect of temperature and humidity on insect DNA integrity evaluated by real-time PCR
Source: J Econ Entomol. 2024 Aug 30;117(5):1995–2002. doi: 10.1093/jee/toae193 (PMC11473036; doi:10.1093/jee/toae193)
Supplement: toae193_suppl_Supplementary_Tables_S1-S5 [file toae193_suppl_supplementary_tables_s1-s5.docx]

**Supplementary Table 1.** Averaged temperature and humidity data for south-east and northern Queensland representing summer (February 2020) and winter (July 2020) conditions. Climate data taken from Australian Bureau of Meteorology (2022). RH = Relative humidity

| **South-east Queensland** | **Northern Queensland** |
| --- | --- |
| **Brisbane**  February: 23–30 °C; RH: 70%  July: 11–22 °C; RH: 66%  Humidity ~50–70% through the year; max > 90%; min ~10–20% | **Cairns**  February: 25–34 °C; RH: 70%  July: 17–26 °C; RH: 70%  Humidity ~60–80% through the year; max > 90%; min ~30% |
| **Moreton**  February: 25–33 °C; RH: 80%  July: 20–28 °C; RH: 80%  Humidity ~60–80% through the year; max > 90%; min ~40% | **Cooktown**  February: 25–33 °C; RH: 80%  July: 19–27 °C; RH: 70%  Humidity ~60-80% through the year; max > 90%; min ~50% |

**Supplementary Table 2.** Average temperature and humidity data recorded in the cabinets during the seven mock tephritid fly treatment experiments. *3B Thermostat failed at 3 weeks; Average temperature weeks 1 to 3 = 34.10 °C ± 0.03°C; Average temperature week 4 = 19.37 °C ± 0.28°C; Humidity was stable.

| Treatment | Set temperature (°C) | Avg. measured temperature (°C) | Set humidity (%) | Avg. measured humidity (%) |
| --- | --- | --- | --- | --- |
| 1 | 20.0 | 19.99 ± 0.04 | 50.0 | 50.17 ± 0.37 |
| 2 | 27.5 | 26.66 ± 0.14 | 70.0 | 71.94 ± 0.95 |
| 3A | 35.0 | 35.25 ± 0.05 | 90.0 | 92.41 ± 1.52 |
| 3B | 35.0 | 26.00 ± 7.33* | 90.0 | 93.15 ± 2.58 |
| 4 | 35.0 | 34.00 ± 0.18 | 50.0 | 51.63 ± 0.62 |
| 5 | 20.0 | 19.33 ± 0.03 | 90.0 | 87.07 ± 5.07 |
| 6 | 25.0-30.0 | 24.94-30.17 | 70.0 | 72.44 ± 0.71 |
| 7 | 22.5-32.5 | 22.11-32.90 | 70.0 | 71.56 ± 5.60 |

**Supplementary Information.** Reaction conditions, efficiency and limit of detection of Dacine-COI PCR assay.

*Methods*

PCR mixtures contained 4.0 μL 5X HOT FIREPol® EvaGreen® HRM Mix (No Rox) (Solis BioDyne, Integrated Sciences, Australia); 0.25 μM of each primer (LCO1490-mod: TYTCAACAAATCATAAAGATATTGG; Dac-COI-r: GTTCAACCTGTACCVGCYCCGTTTTC (Krosch et al. 2020); 2 μL of DNA or lysate; and nuclease-free water up to 20 µL final volume. The assay was run using following conditions: 12 minutes at 95 °C, followed by 40 cycles of 95 °C for 15 seconds, 53 °C for 20 seconds and 72 °C for 20 seconds acquiring to the green (FAM) channel. This was followed by a final melt step ramping from 70–99 °C, rising by 1 °C each step, acquiring to green (FAM/HRM).

Reaction efficiency and limit of detection (LOD) was assessed with the 10-fold dilution series of synthetic Dacine COI gene block starting at 10^7^ copies/ μL down to 10 copies/ μL tested in duplicate. Gene block standards were included in every run at three concentrations ranging from 2x10^4^ to 2x10^6^ copies/reaction allowing the software to automatically calculate DNA copy number in test samples.

*Results*

The calculated reaction efficiency of the Dacine-COI real-time PCR assay was determined to be 84%. Amplification of the synthetic Dacini COI gene block serial dilutions (2x10^7^ – 2x10^1^ copies/ reaction) in this assay generated a corresponding standard curve with a Ct range of 8.4 – 31.4. The assays’ limit of detection was determined to be 20 copies/ reaction based on reactions where the gene block was used as a quantitative measure. The variation between technical replicates was higher than the other species-specific real-time assays. One control sample failed to amplify in this assay (i.e., Ct>40) while the other two controls had technical mean Ct values of 22.35 ± 1.03 and 28.80 ± 1.03, respectively (biological sample mean Ct = 25.57 ± 4.56) (See Supplementary Figure 1).


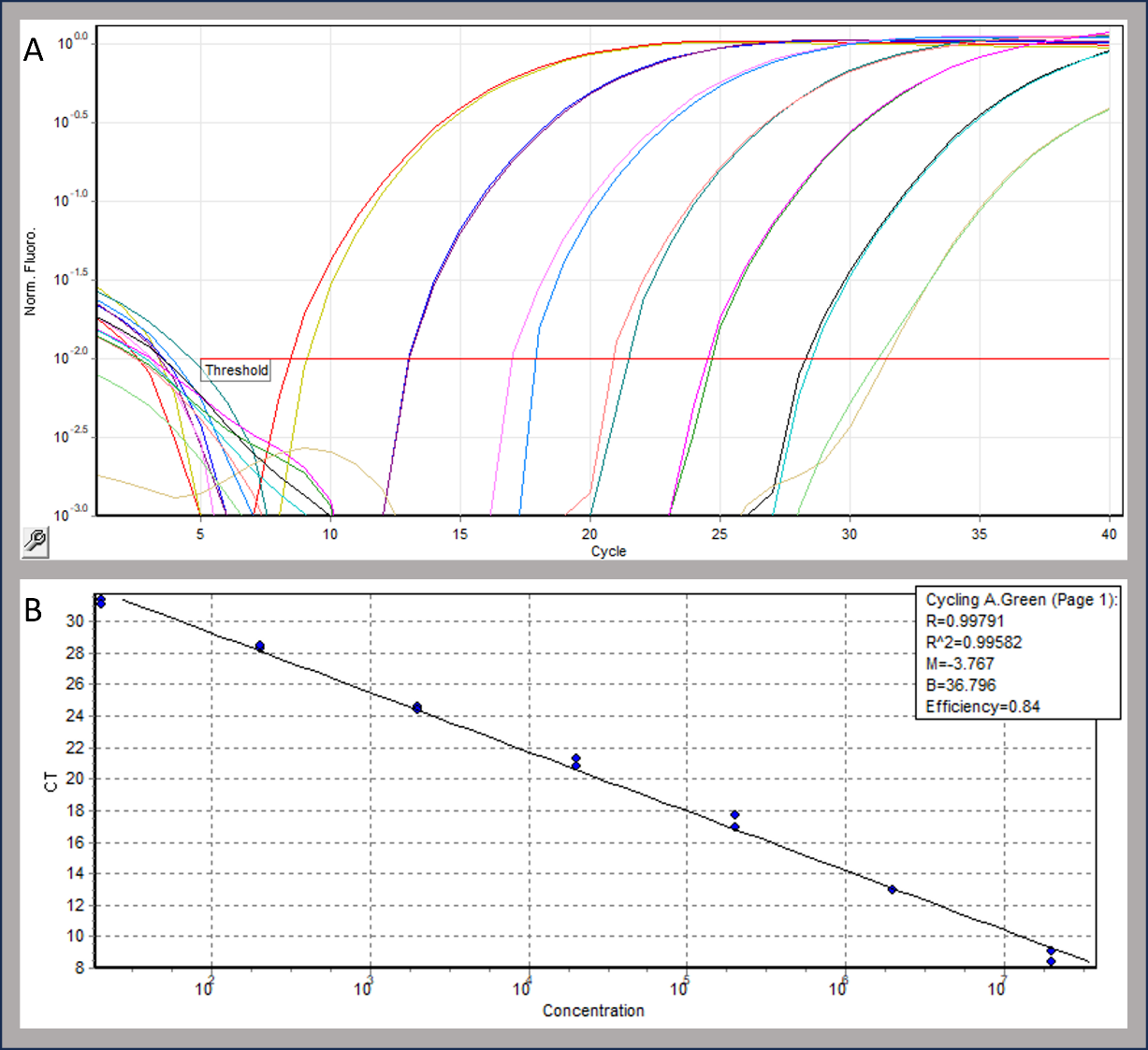


**Supplementary Figure 1.** Evaluation of new Dacini real-time PCR assay. (A) The amplification curves generated by seven concentrations of synthetic Dacini COI gene block, ranging from 2x10^7^ to 2x10^1^ copies/ reaction in 10-fold serial dilutions. Cycle threshold (Ct) is the horizontal red line set at 0.01 Normalised Fluorescence starting at 5 cycles. (B) Standard curve graph showing linear regression analysis of target concentration vs Ct where reaction efficiency (0.84) is determined from the slope (M); R^2^ = 0.996 (correlation coefficient); M is slope; B is intercept.

**Supplementary Table 3.** Gene fragment (gBlock) sequences designed and synthesised and used as positive controls for the three species-specific fruit fly real-time PCR assays and the Dacine-COI assay with GenBank accession numbers for the corresponding COI mitochondrial gene region. Primer binding sites in gBlocks are underlined; probe binding sites are bolded; inserted “ggg” and “ccc” bases are in lowercase.

| Gene fragment name | Sequence 5’-3’ |  | Target species | GenBank accession # |
| --- | --- | --- | --- | --- |
| Btry_qPCR  gBlock | gggAATTGTAACAGCCCATGCTTTCGTAATGATTTTCTTTATAGTTATACCAATTATAATTGGTG**GTTTCGGAAACTGGCT**TGTTCCTTTAATATTAGGTGCGCCCGATATAGCATTCCCACggg |  | *Bactrocera tryoni* | KT864774 |
| Bjar_qPCR  gBlock | gggACAGCTCACGCTTTTGTAATAATTTTCTTTATAGTAATACCTATTATAATCGGTGGTTTCGGAAAT**TGACTAGTTCCTTTAATGTTAGGTG**CCCCCGATATAGCTTTCCCACGAATAAATAATAT  AAGATTTTGGTTATTACCTCCTTCCCTTACACTACTGTTAGTggg |  | *Bactrocera jarvisi* | KT864750 |
| Zcuc_qPCR  gBlock | gggTCGTTTGAGCTGTAGTCCTTACAGCCCTACTATT**ACTTCTATCCCTCCCAGTTCTAGCTGGA**GCTATCACTATGCTTTTAACAGATCGAAATTTAAATACTTCTTTCTTCGACCCAGCTGggg |  | *Zeugodacus cucumis* | JQ420912 |
| Dacini_COI_gBlock | cccTTTCAACAAATCATAAAGATATTGGcccAACTTTATATTTTATCTTCGGAGCCTcccGAGCAGGAATAGTCGGGACATCCCTTcccAGAATTTTAGTCCGGGCTGAACTAcccGGACACCCCGGGGCATTAATCGGAGcccACGATCAGATTTATAATGTAATTGTcccAACAGCCCATGCTTTCGTAATGATTcccTTCTTTATAGTTATACCAATTATAAcccTTGGTGGTTTCGGAAACTGGCTTcccGTTCCTTTAATATTAGGTGCGCCCGAcccTATAGCATTCCCACGAATAAATAATcccATAAGATTTTGATTACTACCCCCTTCCCTTACACTACTcccATTAGTGAGAAGTATAGTAcccGAAAACGGAGCTGGTACAGGTTGAACccc |  | Dacine spp. | MZ520733.1 |

**Supplementary Table 4.** Trap catches, variables and Dacini COI real-time data used in DNA quality comparison with controlled temperature and humidity cabinet data. Two technical replicates tested per sample. If Ct > 40 for all replicates, copy number assumed to be 0. NB: *only one replicate amplified. Climate data is the average recorded by Australian Bureau of Meteorology (2022) for the relevant time period in the field.

| Sample | Date | Location | Time in field | Avg. Temperature (°C) | Avg. Humidity (%) | Total Rainfall (mm) | Trap type | Mean Ct value | Mean Copy number |
| --- | --- | --- | --- | --- | --- | --- | --- | --- | --- |
| Field1 | 26/10/2020 | Brisbane | 2 wks | 27.7 | 52.3 | 86.6 | Lynfield | 39.42* | 0.45 |
| Field2 | 16/11/2021 | Cooktown | 4 wks | 30.8 | 59.6 | 18.2 | Paton | >40 | 0 |
| Field3 | 16/11/2020 | Cooktown | 4wks | 32.3 | 57.3 | 1.8 | Paton | 23.27 ± 5.23 | 4576 |
| Field4 | 16/11/2020 | Cooktown | 4wks | 32.3 | 57.3 | 1.8 | Paton | 20.76* | 3780 |
| Field5 | 17/11/2020 | Cairns | 2 wks | 32.3 | 51.7 | 6.4 | Paton | 27.84 ± 2.69 | 139 |
| Field6 | 24/11/2020 | Brisbane | 2 wks | 29.2 | 52.4 | 0.8 | Lynfield | 25.41 ± 1.13 | 793 |
| Field7 | 8/12/2020 | Brisbane | 2 wks | 30.2 | 52.2 | 4.8 | Lynfield | 26.96 ± 0.33 | 63 |
| Field8 | 14/12/2020 | Cooktown | 4wks | 32.1 | 62.6 | 177.0 | Paton | 30.89 ± 1.66 | 6 |
| Field9 | 14/12/2020 | Cooktown | 4 wks | 32.1 | 62.6 | 177.0 | Paton | >40 | 0 |
| Field10 | 15/12/2020 | Cairns | 2 wks | 32.6 | 60.5 | 26.0 | Paton | 24.17 ± 4.75 | 2128 |
| Field11 | 15/12/2020 | Cairns | 2 wks | 32.6 | 60.5 | 26.0 | Paton | 27.63 ± 7.15 | 571 |
| Field12 | 21/12/2020 | Brisbane | 2 wks | 28.8 | 59.1 | 122.0 | Lynfield | 27.72 ± 0.84 | 386 |
| Field13 | 5/01/2021 | Brisbane | 2 wks | 29.9 | 55.9 | 10.4 | Lynfield | 23.83 ± 4.85 | 2754 |
| Field14 | 5/01/2021 | Brisbane | 2 wks | 29.9 | 55.9 | 10.4 | Lynfield | 18.75 ± 0.22 | 14450 |
| Field15 | 25/01/2021 | Cairns | 2 wks | 30.7 | 72.8 | 178.8 | Paton | 39.29* | 0.02 |
| Field16 | 15/11/2021 | Cairns | 2 wks | 32.6 | 58.5 | 41.0 | Paton | 24.96* | 234 |
| Field17 | 10/11/2021 | Townsville | 2 wks | 34.1 | 58.0 | 12.6 | Lynfield | 21.09* | 3050 |
| Field18 | 16/11/2021 | Cooktown | 4 wks | 30.8 | 59.6 | 18.2 | Paton | 21.15 ± 2.31 | 4852 |
| Field19 | 24/01/2022 | Cairns | 2 wks | 29.3 | 70.9 | 248.8 | Steiner | >40 | 0 |
| Field20 | 10/01/2022 | Cooktown | 4 wks | 31.3 | 61.2 | 160.6 | Paton | 24.08 ± 0.68 | 441 |
| Field21 | 25/01/2022 | Airlie Beach | 2 wks | 30.7 | 58.4 | 35.2 | Steiner | 26.13 ± 2.47 | 2188 |
| Field22 | 24/01/2022 | Cairns | 2 wks | 29.3 | 70.9 | 248.8 | Steiner | 23.17 ± 2.25 | 2660 |
| Field23 | 10/01/2022 | Cooktown | 4 wks | 31.3 | 61.2 | 160.6 | Paton | 23.44 ± 3.49 | 2425 |
| Field24 | 11/01/2022 | Brisbane | 4 wks | 27.2 | 61.6 | 51.8 | Lynfield | 21.82 ± 3.61 | 7110 |
| Field25 | 11/01/2022 | Cairns | 4 wks | 29.7 | 73.4 | 232.0 | Paton | 25.80 ± 4.62 | 702 |
| Field26 | 11/01/2022 | Cairns | 4 wks | 29.7 | 73.4 | 232.0 | Paton | 24.72 ± 5.07 | 13863 |
| Field27 | 11/01/2022 | Cairns | 4 wks | 29.7 | 73.4 | 232.0 | Paton | 22.89 ± 5.15 | 5685 |
| Field28 | 25/01/2022 | Airlie Beach | 2 wks | 30.7 | 58.4 | 35.2 | Steiner | 23.34 ± 0.79 | 7705 |
| Field29 | 8/03/2022 | Cairns | 2 wks | 31.6 | 66.4 | 40.4 | Paton | 23.61 ± 0.81 | 2614 |
| Field30 | 8/03/2022 | Cairns | 2 wks | 31.6 | 66.4 | 40.4 | Steiner | 21.56 ± 1.15 | 2565 |

**Supplementary Table 5.** Adjusted average *Bactrocera tryoni* COI real-time PCR data and calculated copy number for seven treatment groups collected at two time points. Mean Ct value results with the same letter are not significantly different at P = 0.05. Mean copy number results with the same letter are not significantly different at P = 0.05; *one datapoint only for treatment 3 at 2 weeks (3A) as samples failed to amplify (Ct > 40); *cabinet thermostat failed at 3 weeks for treatment 3 at 4 weeks (3B).

| Treatment Group | Temperature (°C) | RH (%) | Mean Ct values | | Mean Copy Number x10^6^/ reaction | |
| --- | --- | --- | --- | --- | --- | --- |
|  |  |  | **2 weeks** | **4 weeks** | **2 weeks** | **4 weeks** |
| 1 | 20.0 | 50 | 16.51^a^ | 16.94^ab^ | 19.29^f^ | 18.32^f^ |
| 2 | 27.5 | 70 | 17.55^b^ | 18.52^cd^ | 13.16^ef^ | 6.31^cd^ |
| 3* | 35.0 | 90 | 19.40^def^ | 20.64^fh^ | 1.31^ab^ | 4.37^bcd^ |
| 4 | 35.0 | 50 | 17.68^bc^ | 20.19^fgh^ | 3.10^abc^ | 0.92^a^ |
| 5 | 20.0 | 90 | 19.67^efg^ | 20.13^fgh^ | 3.38^abc^ | 2.56^abc^ |
| 6 | 25.0-30.0 | 70 | 17.72^bc^ | 18.76^de^ | 8.44^de^ | 5.45^bcd^ |
| 7 | 22.5-32.5 | 70 | 17.34^ab^ | 17.57^b^ | 46.89^g^ | 36.22^g^ |
| Untreated | N/A | N/A | 16.82^ab^ | - | 22.19^f^ | - |
| Pooled Standard Error | | | 0.36 | - | 2.15 | - |

**References**

Australian Bureau of Meteorology. 2022. Climate data online. <http://www.bom.gov.au/climate/data/index.shtml>

Krosch MN, Strutt F, Blacket MJ et al. Development of internal COI primers to improve and extend barcoding of fruit flies (Diptera: Tephritidae: Dacini). Insect Science. 2020:27(143–158. <https://doi.org/https://doi-org.ezp01.library.qut.edu.au/10.1111/1744-7917.12612>.
